# A randomized controlled pilot study of the University of Minnesota mentoring excellence training academy: A hybrid learning approach to research mentor training

**DOI:** 10.1017/cts.2019.368

**Published:** 2019-07-18

**Authors:** Anne Marie Weber-Main, Janet Shanedling, Alexander M. Kaizer, John Connett, Michelle Lamere, Esam E. El-Fakahany

**Affiliations:** 1 Department of Medicine, Medical School, University of Minnesota, Minneapolis, MN, USA; 2 Clinical and Translational Science Institute, University of Minnesota, Minneapolis, MN, USA; 3 Department of Biostatistics and Informatics, University of Colorado, Aurora, CO, USA; 4 Biostatistics Division, School of Public Health, University of Minnesota, Minneapolis, MN, USA; 5 Department of Experimental and Clinical Pharmacology, College of Pharmacy, University of Minnesota, Minneapolis, MN, USA.

**Keywords:** Mentoring, mentor training, online education, hybrid learning, self-paced online course

## Abstract

**Introduction:**

Research mentor training is a valuable professional development activity. Options for training customization (by delivery mode, dosage, content) are needed to address the many critical attributes of effective mentoring relationships and to support mentors in different institutional settings.

**Methods:**

We conducted a pilot randomized controlled trial to evaluate a hybrid mentor training approach consisting of an innovative, 90-minute, self-paced, online module (*Optimizing the Practice of Mentoring, OPM*) followed by workshops based on the *Entering Mentoring (EM)* curriculum. Mentors (*n* = 59) were randomized to intervention or control arms; the control condition was receipt of a two-page mentoring tip sheet. Surveys (pre, post, 3-month follow up) and focus groups assessed training impact (self-appraised knowledge, skills, behavior change) and participants’ perceptions of the blended training model.

**Results:**

The intervention (∼6.5 hours) produced significant improvements in all outcomes, including skills gains on par with those reported previously for the 8-hour EM model. Knowledge gains and intention-to-change mentoring practices were realized after completion of *OPM* and augmented by the in-person sessions. Mentors valued the synergy of the blended learning format, noting the unique strengths of each modality and specific benefits to completing a foundational online module before in-person engagement.

**Conclusions:**

Findings from this pilot trial support the value of e-learning approaches, both as standalone curricula or as a component of hybrid implementation models, for the professional development of research mentors.

## Introduction

As national attention on effective research mentoring in science and medicine disciplines has heightened [1–7], so too has recognition that mentors and mentees can benefit from structured training in how to initiate and sustain high-quality research mentoring relationships [8–11]. A variety of approaches to research mentor development have been reported [12–20], but one of the most widely disseminated [21–23] and well-studied [24–26] models is the *Entering Mentoring (EM)* curriculum [27,28]. These workshops focus on improving mentors’ skills in six competency areas: maintaining effective communication, aligning expectations for the mentoring relationship, assessing mentees’ understanding of research, addressing equity and diversity in mentoring relationships, fostering mentees’ independence, and promoting mentees’ professional development. The curriculum has been adapted for audiences from different disciplines and training stages, with materials publicly available via the Center for the Improvement of Mentored Experiences in Research (www.cimerproject.org). The effectiveness of the 8-hour clinical and translational research adaptation of *EM* [29,30] was demonstrated in a 2010–2011 randomized controlled trial (RCT) [25] conducted across 16 sites, 15 of which had received Clinical and Translational Science Awards (CTSAs) from the National Institutes of Health (NIH). Faculty at our institution, the University of Minnesota, participated in that landmark trial, prompting us to consider how to keep research mentor training a priority and pursue options for expanding its adoption across our campuses.

A major strength of the *EM* training model is its intensive engagement of mentors via case study discussions and other activities that encourage learning and skill building. Two practical limitations to this approach are its time investment and the challenge of scheduling workshops around faculty members’ busy schedules. Thus, when our Clinical and Translational Science Institute (CTSI) was funded in 2011, we began exploring a blended or hybrid approach to mentor training [31–33]: a combination of asynchronous, self-paced, online learning [34–36], followed by a shorter, more focused face-to-face program based on *EM*. We envisioned that the online module – completed at the time and place of participants’ choosing – would complement the *EM* curriculum by providing participants with foundational knowledge about mentoring, prompting reflection on their own practices, and priming them to engage more substantively in the subsequent workshops.

We assembled a team to develop an innovative online module targeted to research mentors of graduate students, fellows, and junior faculty in biomedical, behavioral, and social science fields. The product, *Optimizing the Practice of Mentoring (OPM)*, was launched by our CTSI in 2012 and remains free to users within and outside our institution [37]. Content is organized into five sections: (1) types of mentoring models, (2) research mentor roles and responsibilities, (3) phases of the mentoring relationship, (4) strategies for fostering good relationships, and (5) approaches to addressing mentorship challenges. Learners engage with the material through text, audio, mini presentations, case study explorations, and other brief interactive activities. They also complete a Mentoring Action Plan and have access to an online toolkit. Completion time ranges from 1 to 2 hours, depending on how deeply learners engage with the exercises and explore the supplemental resources.

We packaged *OPM* with a shorter version of the *EM* curriculum to create a hybrid model for research mentor training, which we then offered to the faculty in our Academic Health Center as a new professional development initiative called the *Mentoring Excellence Training Academy (META)*. Launch of the inaugural META in spring 2015 gave us the opportunity to pilot test the hybrid model in a small RCT while acquiring preliminary evaluation data for *OPM*. For this pilot study, the control condition was receipt of a two-page mentoring tips sheet based on *OPM* content. Our research questions were: (1) Could we achieve favorable training outcomes (gains in mentors’ perceived knowledge, skills, and behavior change in comparison to controls) with the hybrid model that were on par with those reported for the more time- and resource-intensive *EM* curriculum? (2) Would participants’ post-training knowledge gains and behavior change be additive for the two META components? (3) From the perspective of participants, is there synergy or unwelcome redundancy across the two components? And how might the META model be improved? We report our findings here, including data gathered from online surveys and focus groups.

## Materials and Methods

### Design

We conducted a single-site, two-arm, pilot RCT at the University of Minnesota Academic Health Center, Twin Cities Campus. The protocol was reviewed and approved by the University of Minnesota Institutional Review Board prior to recruitment and data collection.

### Recruitment and Eligibility Criteria

We recruited faculty participants in March 2015 through email announcements sent by the associate deans for research in our Academic Health Center’s six schools and colleges: Medical School, School of Nursing, College of Pharmacy, School of Public Health, College of Veterinary Medicine, and School of Dentistry. The email directed recipients to an online eligibility survey that screened for our inclusion criteria: (1) currently holds a faculty appointment, (2) has not previously completed the online module used in the META, (3) has at least one year of experience mentoring graduate students, postdoctoral fellows, or junior faculty members in a research setting, and (4) is mentoring at least one person in a research setting for the next 3 months or longer. As this was a pilot study, we did not compute an *a priori* power calculation; rather, we included all interested faculty who met our inclusion criteria. Our enrollment target (maximum of 60 study participants, 30 per group) was based on practical considerations that included our limited time frame for recruitment (1 month) and the maximum number of faculty (25–30) we could accommodate in one cycle of the workshops for those randomized to the intervention arm.

### Randomization and Blinding

Eligible participants were ranked by the number of people they had previously mentored in research. We assessed this at enrollment as an approximation of prior mentoring experience. Within consecutive pairs on the ranked list, one person was randomly assigned to the intervention group and the other to the control group. Participants were not blinded to treatment condition. Those randomized to the control group were given priority registration for the next META offering.

### Intervention Condition

Participants in the intervention group experienced the two-component META professional development program from April to May 2015. The first component was completion of the online module *OPM*. The second was participation in two in-person workshops (5 hours total, 5–10 participants each). Specifically, we implemented an adapted version of the 8-hour *EM* curriculum *Mentor Training for Clinical and Translational Researchers*, focusing on five competency domains addressed in the following sequence: workshop 1 covered maintaining effective communication, establishing and aligning expectations, and addressing equity and diversity; workshop 2 addressed fostering independence and promoting professional development. Both workshop sessions were facilitated by two of the authors who were trained to deliver the curriculum during its testing in the national multisite RCT.

### Control Condition

Participants in the control group received a two-page mentoring tip sheet (Supplemental Material) that summarized core content from *OPM*. This resource included a definition of research mentoring, a brief explanation of different mentoring models, and succinct descriptions of mentor responsibilities, phases of a mentoring relationship, and strategies for building healthy mentoring relationships.

### Outcome Measures and Data Collection

We collected data through online surveys (REDCap software) and focus groups. Participants received a mentoring resource book (∼$25 value) upon completion of the final follow-up survey.

#### Surveys

Baseline surveys assessed participants’ demographic characteristics, professional background, mentoring experience, mentoring knowledge (9 items, 5-point Likert-type scale from 1 = not at all knowledgeable to 5 = extremely knowledgeable), and mentoring skills (12 items, 5-point Likert-type scale from 1 = not at all skilled to 5 = extremely skilled). We developed knowledge items that reflected the META’s core content and learning objectives (e.g., “Range of mentoring functions I am expected to perform,” “Steps I can take at the beginning of a mentoring relationship to create a good foundation,” “Ways that diversity can influence mentor-mentee interactions,” “Pros and cons of mentoring models that I should be aware of in my own mentoring practice”). For control participants, we assessed knowledge at baseline and after receiving the tip sheet (posttest 1 survey). For intervention participants, we assessed knowledge at baseline, once after completing just the online module (posttest 1 survey) and again after completing the in-person facilitated sessions (posttest 2 survey) to capture potential additive gains.

Our second quantitative outcome was perceived skills gains from baseline to 3-month follow-up. We selected this time point to give participants time to reflect on and apply their training with current mentees. In previous research on the full 8-hour *EM* curriculum [25], participants reported statistically significant improvements in their self-reported mentoring skills, assessed via the validated 26-item Mentoring Competency Assessment (MCA) [38]. With this key outcome well established, we created a smaller and more tailored set of skills items for our pilot study of the META. First, we selected items from the MCA that best aligned with the subset of mentor competencies – and specific learning objectives within each competency domain – that we covered in our customized training program (e.g., “Working effectively with mentees with backgrounds different from mine,” “Negotiating with my mentees a path to their professional independence”). Second, we created new items to reflect content unique to the online module (e.g., “Bringing appropriate closure to my mentoring relationships,” “Striking a balance between issuing challenges and offering support”).

As our third quantitative outcome, we evaluated the proportion of participants in each study group who indicated an intention to change their mentoring behaviors at post-intervention, and the proportion who reported having actually implemented changes in their mentoring behaviors at 3-month follow-up. Response options were *yes*, *not yet but considering*, or *no*.

The remaining post-survey items (closed and open-ended) evaluated participants’ satisfaction with the training. We specifically probed their perceptions of the META’s value as individual components and as a packaged hybrid curriculum.

#### Focus groups

To more deeply assess participants’ experiences with the training, we invited intervention group participants to participate in a 1-hour focus group conducted by a hired facilitator. We scheduled three focus groups of five to six faculty each. Given several no-shows, we ultimately conducted two groups: one with four participants and one with three. With only one person showing up for the third group, the facilitator conducted this as a semi-structured interview. Discussions were audio recorded and transcribed. Question prompts were designed to ascertain participants’ perceptions of the META’s value:

1. What influenced your decision to participate in the META?
2. In what way(s) has your participation influenced your understanding of effective mentoring?
3. In what way(s) has your participation influenced your mentoring practices?
4. How well do you feel the specific components of the META worked together as a package?
5. What was most valuable to you about: The META overall? The online self-study module? The face-to-face facilitated group discussions?
6. How do you think the META might be improved or enhanced?
7. What would you say to a colleague who tells you she has been asked to participate in the next META offering?
8. What else would you like to share about the META, mentoring in general, or mentoring at the University of Minnesota?

### Analyses

We hypothesized that knowledge gains, skills gains, and changes in mentoring practices would be greater for intervention participants than control participants and that knowledge and behavior changes would increase to a greater extent after completion of both training components (online module + facilitated group sessions). The two-sample *t*-test was used to determine if there were significant differences between the intervention and control groups in change from baseline to posttest knowledge scores and change from baseline to 3-month follow-up skills scores. The paired version of the *t*-test was used for analyses comparing changes within the intervention group at different survey times. Given the pilot nature of our study, we did not adjust *P*-values for multiple comparisons. When responses were missing within each survey, these values were excluded from analyses (i.e., no imputation was used to fill in missing data). In addition to analyzing individual knowledge and skills items, we created non-validated composite measures for the 9 knowledge items and 12 skills items. We used the average score across all items rather than the sum of all individual scores in our analyses to maintain the same scale as the individual questions and facilitate easier comparisons.

Focus group transcripts were manually mined for keywords and phrases, which were then entered into concept mapping software (MindMap 2.0). Words and statements with similar intent were connected within the tool and the number of incidents or people agreeing with the statement during discussion were recorded. Initial clusters were generated for repeated elements. These clusters were then reviewed for emerging themes by two of the authors not directly involved in implementing the intervention.

## Results

### Participants

**Fig. 1.**
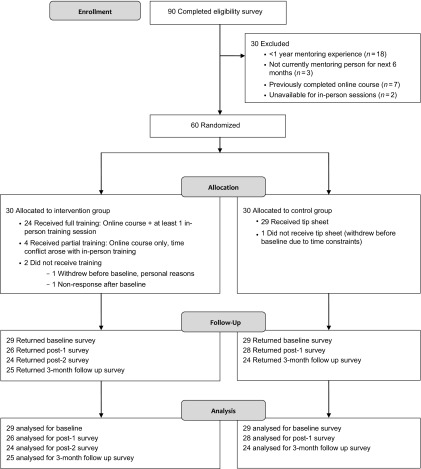
CONSORT flow diagram. The study was a pilot randomized trial of a hybrid training intervention versus control condition (receipt of mentoring tip sheet) to assess the intervention’s impact on mentoring knowledge, skills, and behavior change.

A participation flow diagram is presented in Fig. 1. Of the 90 faculty members screened, 60 met the inclusion criteria and were randomized. One participant withdrew before data collection commenced. Baseline characteristics for the remaining 59 participants are summarized in Table 1. Participants were predominantly white, non-Hispanic and non-Latino, but more diverse in sex and age. The majority (64%) were faculty from the Medical School, the largest of our AHC schools and colleges. Although many were assistant professors (44%) and had limited (1–5 years) mentoring experience (42%), our sample included faculty at higher ranks and with a wide range of mentoring experience. Thirteen (22%) participants indicated they had previously participated in some form of mentor training.

**Table 1.** Baseline characteristics of pilot study participants

Mentor Characteristics	Intervention	Control
	(*N* = 29)	(*N* = 30)
Sex, no. (%)		
Female	13 (44.8)	18 (60.0)
Male	15 (51.7)	11 (36.7)
Missing	1 (3.4)	1 (3.3)
Age, no. (%)		
31–40 years	11 (37.9)	6 (20.0)
41–50 years	6 (20.7)	13 (43.3)
51–60 years	10 (34.5)	7 (23.3)
61–70 years	1 (3.4)	2 (6.7)
> 70 years	0 (0.0)	1 (3.3)
Missing	1 (3.4)	1 (3.3)
Race^a^, no. (%)		
White	25 (86.2)	26 (86.7)
Asian	1 (3.4)	3 (10.0)
Two or more races/ethnicities	1 (3.4)	0 (0.0)
Unknown or missing	2 (6.9)	1 (3.3)
Hispanic or Latino ethnicity, no. (%)	2 (6.9)	0 (0.0)
School or college, no. (%)		
Medical	19 (65.5)	19 (63.3)
Veterinary medicine	5 (17.2)	2 (6.7)
Pharmacy	2 (6.9)	3 (10.0)
Dentistry	1 (3.4)	2 (6.7)
Nursing	1 (3.4)	3 (10.0)
Public health	1 (3.4)	1 (3.3)
Faculty rank, no. (%)		
Assistant professor	16 (55.2)	10 (33.3)
Associate professor	7 (24.1)	10 (33.3)
Professor	6 (20.7)	10 (33.3)
Years of experience being a research mentor, no. (%)		
1–5 years	14 (48.3)	11 (36.7)
6–10 years	5 (17.2)	7 (23.3)
11–15 years	4 (13.8)	2 (6.7)
16–20 years	2 (6.9)	4 (13.3)
> 20 years	4 (13.8)	5 (16.7)
Had previous mentorship training, no. (%)	4 (13.8)	9 (30.0)
Hours of previous mentor training, mean (SD)	7.0 (2.45)	14.7 (18.3)
Types of trainees currently mentoring, no. (%)		
Junior faculty	16 (55.2)	17 (56.7)
Postdoctoral fellows	12 (41.4)	13 (43.3)
Clinical fellows	9 (31.0)	11 (36.7)
PhD/Master’s students	13 (44.8)	18 (60.0)
Medical or other health care professional students	19 (65.5)	18 (60.0)
Undergraduate students	12 (41.4)	17 (56.7)
High school students	1 (3.4)	8 (26.7)
Recipients of NIH Mentored Career Development Awards	1 (3.4)	2 (6.7)
Participants in NIH T32 programs	6 (20.7)	8 (26.7)
Research focus^b^, no. (%)		
Behavioral	6 (20.7)	5 (16.7)
Clinical	10 (34.5)	17 (56.7)
Community-engaged	7 (24.1)	1 (3.3)
Educational	11 (37.9)	8 (26.7)
Field/applied	8 (27.6)	5 (16.7)
Lab-based	8 (27.6)	6 (20.0)
Theoretical	1 (3.4)	1 (3.3)
Translational	7 (24.1)	10 (33.3)

SD, standard deviation.

a Race and ethnicity were self-reported. Other response options were American Indian or Alaskan Native, Black or African American, Native Hawaiian or other Pacific Islander; no participants self-identified in these groups.

b Participants could report more than one research focus area.

### Training Impact

**Fig. 2.**
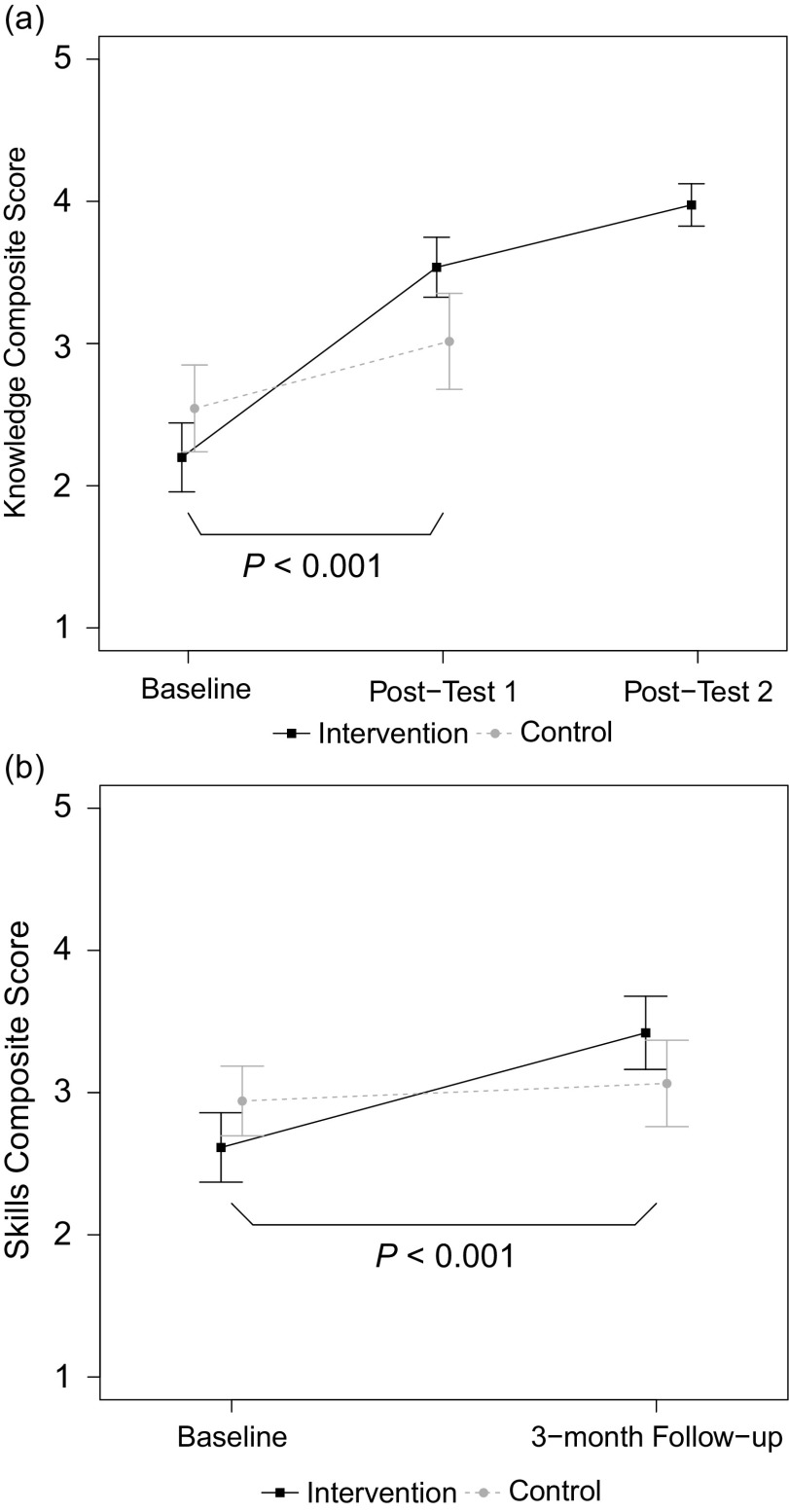
Self-reported changes in mentors’ knowledge and skills. Group comparisons of mean composite scores across study time points for (a) mentoring knowledge and (b) mentoring skill. Group means are shown with 95% confidence intervals. *P* values are for *t*-tests of group differences in the indicated change.

All intervention participants reported spending a minimum of 1 hour engaging with the online module content; 32% spent 1–1.5 hours, 48% 1.5–2 hours, and 20% over 2 hours. After completion of the online module (posttest 1), intervention participants reported greater gains in mean [SD] composite knowledge score than control participants (+1.34 [0.71] vs. +0.51 [0.71], *P* < 0.001; Fig. 2a). We observed an additional smaller but significant increase in composite knowledge for intervention participants after they completed both components of the training program (+0.35 [0.36] from posttest 1 to posttest 2, *P* = 0.001). Findings were similar when change scores for knowledge items were analyzed individually. Intervention participants exhibited greater knowledge gains than control participants for eight of the nine items at post 1 (*P* < 0.05; Table 2) and for all items at posttest 2. The one knowledge item that increased but did not reach significance until posttest 2 was “Knowledge of specific strategies I can apply to maintain effective relationships and address challenges.”

**Table 2.** Summary of knowledge item scores by group for each survey, changes from baseline to posttest 1, and group differences in changes from baseline

Item	Survey	Control		Intervention		Group Difference in Mean Change from Baseline	*P*-value^b^
		Survey Mean (SD)^a^	Mean Change from Baseline (95% CI)	Survey Mean (SD)^a^	Mean Change from Baseline (95% CI)	(Intervention- Control, 95% CI)	
1. Range of mentoring functions (roles, responsibilities) I am expected to perform	Baseline	3.03 (0.82)	–	2.55 (0.83)	–		
	Posttest 1	3.22 (0.85)	0.22 (−0.11, 0.56)	3.65 (0.69)	1.08 (0.78, 1.38)	0.86 (0.42,1.29)	<0.001
	Posttest 2	–	–	4.00 (0.51)	1.42 (1.17, 1.66)		
2. Steps I can take at the beginning of a mentoring relationship to create a good foundation	Baseline	2.79 (0.94)	–	2.62 (0.86)	–		
	Posttest 1	3.04 (0.76)	0.26 (−0.08, 0.60)	3.69 (0.68)	1.04 (0.71, 1.37)	0.78 (0.32,1.24)	0.003
	Posttest 2	–	–	4.17 (0.48)	1.50 (1.11, 1.89)		
3. Strategies I can apply to maintain effective mentoring relationships and address challenges	Baseline	2.55 (0.87)	–	2.38 (0.82)	–		
	Posttest 1	3.15 (0.91)	0.63 (0.26, 1.00)	3.58 (0.76)	1.15 (0.73, 1.58)	0.52 (−0.02,1.07)	0.06
	Posttest 2	–	–	3.96 (0.47)	1.52 (1.16, 1.89)		
4. Value of using individual development plans with mentees	Baseline	2.79 (1.05)	–	2.21 (1.08)	–		
	Posttest 1	3.15 (0.99)	0.33 (−0.08, 0.74)	3.54 (0.86)	1.35 (0.88, 1.82)	1.02 (0.4,1.62)	0.002
	Posttest 2	–	–	4.21 (0.66)	1.96 (1.52, 2.40)		
5. Ways that diversity can influence mentor-mentee interactions	Baseline	2.34 (1.17)	–	2.10 (0.94)	–		
	Posttest 1	2.85 (0.99)	0.59 (0.14, 1.05)	3.50 (0.76)	1.42 (1.01, 1.84)	0.83 (0.23,1.43)	0.008
	Posttest 2	–	–	3.92 (0.50)	1.75 (1.26, 2.24)		
6. Specific biases and prejudices that might influence my approach to mentoring	Baseline	2.52 (1.15)	–	2.17 (0.85)	–		
	Posttest 1	2.93 (0.92)	0.41 (−0.02, 0.84)	3.52 (0.65)	1.36 (0.90, 1.82)	0.95 (0.34,1.57)	0.003
	Posttest 2	–	–	3.88 (0.61)	1.63 (1.18, 2.07)		
7. Value of and methods for fostering professional development toward independence	Baseline	2.66 (1.01)	–	2.24 (0.87)	–		
	Posttest 1	2.93 (0.87)	0.33 (−0.06, 0.73)	3.44 (0.65)	1.28 (0.89, 1.67)	0.95 (0.41,1.49)	0.001
	Posttest 2	–	–	3.92 (0.58)	1.67 (1.22, 2.11)		
8. Pros and cons of mentoring models that I should be aware of in my own mentoring practice	Baseline	2.21 (1.15)	–	1.76 (0.83)	–		
	Posttest 1	2.88 (1.11)	0.77 (0.35, 1.19)	3.31 (0.88)	1.58 (1.02, 2.14)	0.81 (0.12,1.49)	0.022
	Posttest 2	–	–	3.58 (0.78)	1.79 (1.29, 2.29)		
9. Sources of mentoring info, resources, and tools that I can use in my mentoring practice	Baseline	2.00 (0.96)	–	1.76 (0.79)	–		
	Posttest 1	2.74 (1.10)	0.61 (0.25, 0.97)	3.72 (0.74)	1.96 (1.52, 2.40)	1.35 (0.8,1.9)	<0.001
	Posttest 2	–	–	4.00 (0.67)	2.22 (1.81, 2.63)		

a Items scored on a 5-point Likert-type scale: 1 = not at all knowledgeable, 2 = somewhat knowledgeable, 3 = moderately knowledgeable, 4 = very knowledgeable, 5 = extremely knowledgeable.

b Two-sample *t*-tests for mean difference of change in item score by group (*P* values not corrected for multiple comparisons).

For mentoring skills analyzed as a composite score, the change in mean [SD] score from baseline to 3-month follow up was greater in the intervention group than in the control group (+0.85 [0.70] vs. +0.17 [0.53], *P* < 0.001; Fig. 2b). When change scores for skills items were analyzed individually, intervention participants exhibited greater gains than control participants for 9 of 12 items (*P* < 0.05; Table 3). Exceptions were “Communicating effectively with my mentees,” “Helping my mentees articulate focused career goals,” and “Bringing appropriate closure to my mentoring relationships,” for which group differences were not significant.

**Table 3.** Summary of skills item scores by group for each survey, changes from baseline to 3-month follow-up, and group differences in changes from baseline

Question	Survey	Control		Intervention		Group Difference in Mean Change from Baseline	*P*-value^b^
		Survey Mean (SD)^a^	Mean Change from Baseline (95% CI)	Survey Mean (SD)^a^	Mean Change from Baseline (95% CI)	(Intervention- Control, 95% CI)	
1. Communicating effectively with my mentees	Baseline	3.32 (0.72)	–	3.14 (0.76)	–		
	3-Month	3.38 (0.71)	0.13 (−0.17, 0.43)	3.50 (0.66)	0.35 (−0.15, 0.85)	0.22 (−0.35,0.79)	0.443
2. Working with my mentees to identify and align expectations	Baseline	2.93 (0.84)	–	2.74 (0.76)	–		
	3-Month	2.96 (0.88)	0.04 (−0.29, 0.38)	3.48 (0.71)	0.74 (0.27, 1.21)	0.70 (0.13,1.26)	0.017
3. Assessing my mentees’ knowledge and skills	Baseline	3.00 (0.85)	–	2.57 (0.96)	–		
	3-Month	3.04 (0.77)	0.09 (−0.17, 0.34)	3.28 (0.68)	0.75 (0.31, 1.19)	0.66 (0.17,1.16)	0.01
4. Helping my mentees articulate focused career goals	Baseline	3.00 (0.93)	–	2.57 (0.88)	–		
	3-Month	3.42 (0.88)	0.42 (0.04, 0.79)	3.56 (0.71)	1.00 (0.52, 1.48)	0.58 (−0.01,1.18)	0.054
5. Fostering my mentees’ confidence and scientific creativity	Baseline	2.90 (0.94)	–	2.75 (0.89)	–		
	3-Month	3.00 (0.83)	0.17 (−0.13, 0.46)	3.48 (0.87)	0.75 (0.33, 1.17)	0.58 (0.08,1.08)	0.023
6. Striking a good balance between issuing challenges and offering support	Baseline	2.72 (0.80)	–	2.61 (0.83)	–		
	3-Month	2.96 (0.95)	0.29 (−0.11, 0.69)	3.40 (0.82)	0.88 (0.52, 1.23)	0.59 (0.06,1.11)	0.03
7. Negotiating with my mentees a path to their professional independence	Baseline	2.66 (0.81)	–	2.26 (0.76)	–		
	3-Month	2.67 (0.96)	0.04 (−0.34, 0.43)	3.20 (0.96)	1.00 (0.50, 1.50)	0.96 (0.34,1.58)	0.003
8. Recognizing and respecting individual differences in my mentees	Baseline	3.57 (0.74)	–	3.18 (0.82)	–		
	3-Month	3.38 (0.71)	−0.17 (−0.48, 0.14)	3.84 (0.85)	0.67 (0.24, 1.09)	0.84 (0.33,1.35)	0.002
9. Working effectively with mentees with backgrounds different from mine	Baseline	3.07 (1.00)	–	2.75 (1.00)	–		
	3-Month	3.04 (0.86)	0.00 (−0.45, 0.45)	3.64 (0.86)	1.00 (0.53, 1.47)	1.00 (0.37,1.63)	0.003
10. Overcoming challenges that arise in my mentoring relationships	Baseline	2.66 (0.86)	–	2.32 (0.77)	–		
	3-Month	2.88 (0.95)	0.25 (−0.19, 0.69)	3.40 (0.82)	1.13 (0.67, 1.58)	0.87 (0.26,1.49)	0.006
11. Bringing appropriate closure to my mentoring relationships	Baseline	2.31 (0.97)	–	2.29 (0.94)	–		
	3-Month	2.75 (0.99)	0.54 (0.06, 1.02)	3.00 (0.93)	0.83 (0.40, 1.25)	0.29 (−0.34,0.91)	0.365
12. Self-reflecting on my own mentoring practices	Baseline	2.72 (0.75)	–	2.29 (0.90)	–		
	3-Month	3.08 (1.06)	0.38 (−0.02, 0.77)	3.52 (0.71)	1.29 (0.89, 1.69)	0.91 (0.37,1.46)	0.001

a Items scored on a 5-point Likert-type scale: 1 = not at all skilled, 2 = somewhat skilled, 3 = moderately skilled, 4 = very skilled, 5 = extremely skilled.

b Two-sample *t*-tests for mean difference of change in item score by group (*P* values not corrected for multiple comparisons).

**Fig. 3.**
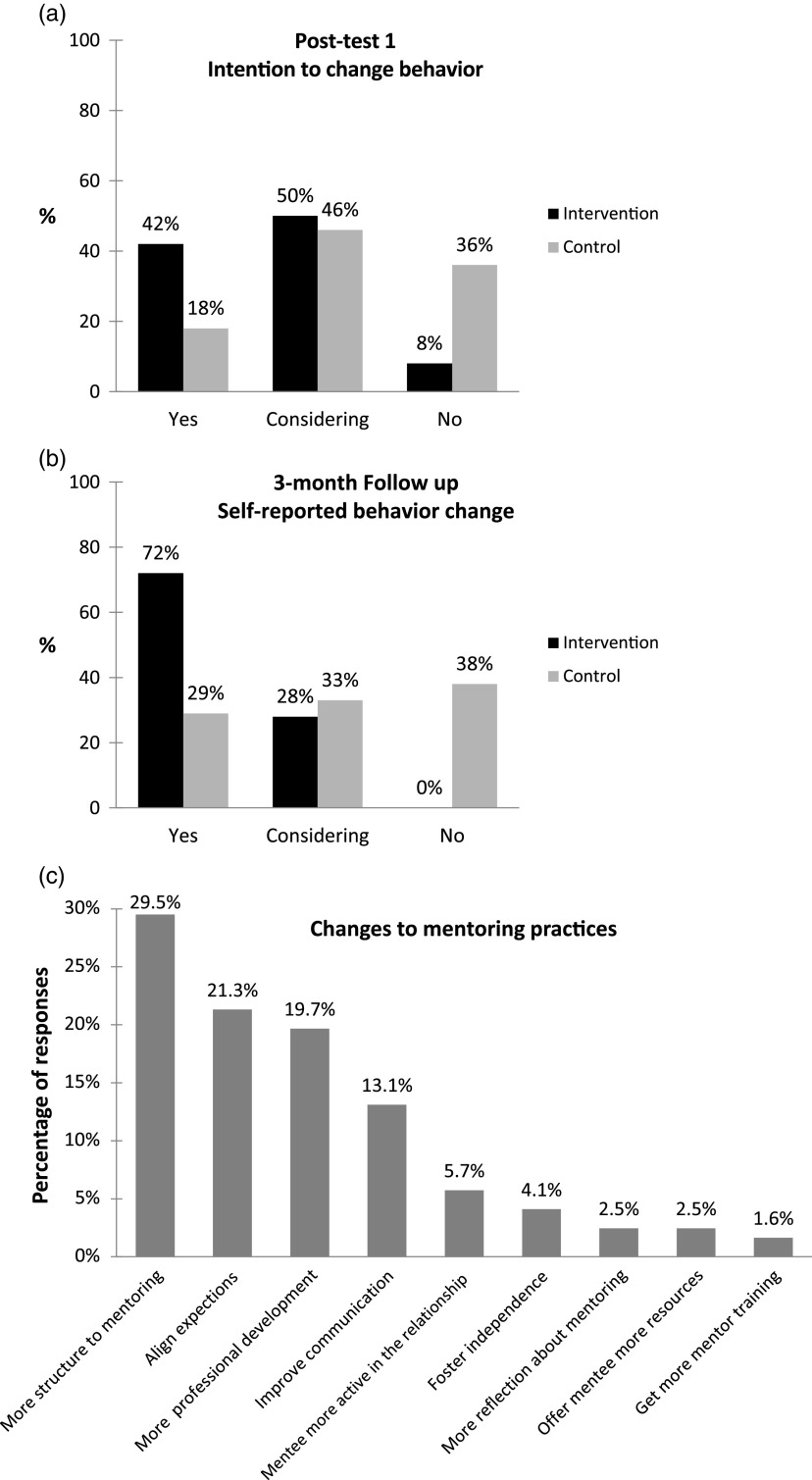
Self-reported changes in mentoring practices. (a) Group comparisons of participants’ intentions to change their mentoring behaviors at posttest 1 (immediately after training or receipt of tips sheet). (b) Group comparisons of participants’ self-reported behavior change at 3-month follow-up. (c) Types of behavior changes planned or implemented by intervention participants at posttest 1, posttest 2, and 3-month follow up (*n* = 122 total responses).

After completing *OPM* (posttest 1), 42% (11/26) of intervention participants noted they planned to change their mentoring practices as a result of the online training versus 18% (5/28) of control participants (Fig. 3a). This proportion increased to 100% (24/24) for the intervention group after completion of the facilitated workshops (data not shown). At 3-month follow-up, 72% (18/25) of intervention participants reported having implemented a change in their mentoring practices versus 29% (7/24) of control participants (Fig. 3b).

In open-ended survey questions, we asked respondents to define the behavior changes they were either thinking about, intending to implement, or had successfully put into practice. Intervention participants offered a total of 122 comments across the three surveys. When we examined and coded comments by theme (Fig. 3c), the most frequently cited behavior change (∼30% of responses) was to adopt a more structured, intentional approach to their mentoring (e.g., “The mentoring process is now more structured, with clearly defined goals that go beyond the day-to-day work.”). Also common were devoting more attention to aligning expectations at the beginning of a mentoring relationship (e.g., “I have concentrated more on discovering and balancing expectations on both sides. I have also tried to direct the relationship more on [my mentees’] expressed needs and less on my perception of those needs.”), putting greater effort into understanding and supporting their mentee’s individual development goals (e.g., “I have had a conversation with my post-doc about her career goals and made a plan with her to achieve them.”), and taking steps to reflect on and improve their communication with mentees (e.g., “[I’ve had] more open discussion of obstacles”).

### Perceived Value of Training

#### Survey results

A majority of participants agreed or strongly agreed that the META was a valuable use of their time (Table 4). Although perceived value was higher for the facilitated group sessions than for the online module (96% vs. 69%, respectively), 75% of participants indicated that they would recommend the full hybrid model to their colleagues versus 25% who would recommend just the in-person workshops. As other indicators of the hybrid curriculum’s value, 79.5% (17/24) of respondents strongly disagreed or disagreed that the two components “were redundant,” and 96% (18/24) strongly agreed or agreed that “the facilitated group sessions successfully built on the knowledge I gained from the online module.”

**Table 4.** Value of the Mentoring Excellence Training Academy (individual components and full hybrid model)

Item	*N*	Strongly Agree or Agree (%)	Neither Agree nor Disagree (%)	Disagree or Strongly Disagree (%)
1. Participating in the facilitated group sessions was a valuable use of my time	24	96	4	0
2. Participating in the online module was a valuable use of my time	26	69	27	0
3. The online module provided me with important knowledge and resources on mentoring	24	84	12	4
4. I benefited from the opportunity to learn from other mentors in the facilitated group sessions	24	96	4	0
5. The facilitated group sessions successfully built on the knowledge I gained from the online module	24	96	4	0
6. The online module and facilitated group sessions were redundant	24	8	12.5	79.5

We asked in posttest 2: Initially, how did you feel about taking the time to complete the META? and Now, having completed the training, did those feelings change? If so, how? The two most common initial perceptions were a sense of enthusiasm (e.g., “Eager anticipation,” “Very interested in learning ways to improve my mentoring”) and concern about the time commitment the program would require (e.g., “Apprehensive about investing so much time,” “Mixed, big commitment,” “Open to learning yet always a challenge finding time”). Having completed the META, respondents uniformly expressed that the time was well spent (e.g., “Exceeded expectations, it was a worthwhile investment of time;” “[My] feelings changed; it was well worth the time,” “I was very pleasantly surprised by how useful the training was; it was orders of magnitude better than most training sessions we attend”).

#### Focus group results

For the eight mentors who participated in focus groups or semi-structured interviews, comments clustered into four primary themes: (1) perceptions of the training’s value, (2) insights acquired, (3) suggestions for improvement, and (4) reflections on the institutional climate for mentoring.

1. Perceptions of META’s value. When asked how well the multimodal training worked as a package, all participants expressed appreciation for how the components complemented one another. For example:
  “The online module lined up all the basics we need to learn…. then the face-to-face, we saw a lot of different cases, so that’s actually very useful to us, because most of them are applicable to me, to the real situation I’m in. So, I think that was the perfect combination, the theory and the practice.”
  “I thought that the in-person complemented the online pretty well. It kind of covered the same topics, but then you got the chance to sort of practice them.”
  “I really like the dual approach, because, I think, with the different methods, you can approach different learners in a different way. And I personally needed both…”
  Participants noted it was useful to complete the online module before the facilitated small group training, because it provided a common framework and helped them feel more prepared for and comfortable with the workshop sessions:
  “It was definitely helpful to have that component [online module] ahead of the face-to-face meetings… initially I really had no idea what was going to happen or what we were going be looking at… but when I got [to] the online portion it kind of helped me feel more comfortable being involved in a face-to-face. I remember feeling relieved at understanding what we were going to be doing a little bit more.”
  “I think what was most valuable about it [online module] was that it actually helped to enhance some of the discussion that you had with other people who were sitting at the table.”
  “It’s good to have everybody on the same page to start with, so having an online component so everybody is talking in the same language, I think that’s good to set the pace.”
  “The two, the online and the actual workshops, worked well together. And I think you need the online first to ground us all.”

For the online module, participants said they appreciated the numerous references and resources that it provided. As one mentor explained, it was useful to realize that “people have studied this [mentoring]; they know effective ways to do that. And they have tools. You don’t have to create things from scratch.” As weaknesses, some participants thought the amount of online content became overwhelming and wanted more time to explore these materials before starting the workshops:

“I remember not having a lot of time to complete the online training, and feeling overwhelmed because there was a lot. It wasn’t so much getting to the content, but it was all the resources.”

The workshops were highly regarded by all focus group participants. For some, group discussions helped to validate their current mentoring practices, while others were challenged to rethink their approaches. All appreciated hearing about other mentors’ experiences and having the opportunity to build new relationships with other mentors: “The sharing of stories was by far the most valuable… because I got not only to hear other people’s perspectives but there’s certain parallels that you can draw, depending on your practice site or your situation, that are kind of universal.”

1. Key mentoring insights acquired. Participants reported a diverse array of takeaways from the training. As shown by the sample quotations in Table 5, these insights are directly reflective of at least one of the training’s learning objectives, offering additional evidence of the program’s effectiveness.
2. Suggestions for improvement. Participants offered thoughtful suggestions for enhancing the META. One recommendation was to break up the online material, with participants completing *OPM* in smaller portions at defined time points before or between workshops. Adding short homework assignments was suggested as a way to reinforce content. The remaining feedback focused on sustainability – how to maintain participants’ enthusiasm, encourage application of content, and promote broader dissemination of mentoring tools and evidence-based practices. There was interest in knowing which faculty within a specific school or college had completed the META so as to facilitate networking among those committed to high-quality mentoring. Other ideas were to bring in speakers on mentoring topics or to conduct follow-up workshops that include peer coaching to resolve mentoring dilemmas. Last, participants recommended creating a centralized repository for the sharing of mentoring resources.
3. Reflections on local mentoring climate. Most of the detailed comments for this theme were specific to our academic health center, and thus are not presented here. However, there was consensus on a few issues with broader relevance: (1) that mentor training is valuable and should be encouraged; (2) that institutions wanting to be world class need to take a critical look at the quality of their mentoring; and (3) that mentorship is a critical academic activity that needs to be valued by institutions and given meaningful credit.

**Table 5.** Examples of insights acquired by participants from the Mentoring Excellence Training Academy

Mentoring Insights	Illustrative Focus Group Responses
Applying different mentoring models	“We decided to go from having the one-on-one mentor, like an academic advisor, to allowing our new residents to pick, in addition to their assigned person, two other individuals to be on a mentoring team.”
Aligning expectations	“I think the expectations piece… was one part that I don’t think I had really considered. Which sounds silly, but when you think about it, you know, I think we come to everything with our own expectations, but often aren’t taking into account our mentees’ expectations and balancing those.”
Being culturally aware, communicating effectively	“I also thought the discussion of cultural pieces was very valuable. Probably something we don’t think about very often, so that was very helpful for me.” “I remember, during our session, talking about a particular student that I was mentoring…. it’s a student who is from a different country, and I was struggling with that difference, and just being patient. We talked about…resolving conflicts, and being sensitive to cultural differences. And I think that the training sort of helped me to put that into perspective and to just focus more on the relationship building. And to be upfront about when I was uncomfortable [or] I didn’t understand.”
Promoting professional development	“I think for us, the biggest thing that we’re working to implement is…the development plans. So, in one of the workshops where we kind of went through some of the options for that, there were fantastic templates…. So, kind of taking those examples and building one that fits our program.”
Mentoring for different developmental stages	“The idea of self-stages of mentorship….a faculty member needs certain things, and she needs something very different five or ten years down the line.”
Broader view of a mentor’s roles and different mentoring practices, tools.	“The Academy kind of helped me push the boundaries of what’s defined as being a mentor – different ways, different things, giving tools. Kind of like a little circle that you’re pushing the borders out, making it bigger, and then getting tools to help fill in those areas that you just added. So, it was useful for me to see a bigger universe than what I’d been thinking of.”

## Discussion

Our pilot study results demonstrate the beneficial impact of training research mentors via a hybrid approach – one that pairs an innovative, self-paced, online learning module (*OPM*) with facilitated in-person workshops modeled on the *EM* curriculum. Participants appreciated the sequencing of the META’s two components, noting that *OPM* provided them with fundamental information about research mentoring while introducing them to relevant resources and preparing them to engage in substantive conversations with other mentors during the subsequent face-to-face workshops.

After completing just *OPM*, META participants had significant improvements in self-appraised knowledge and intention to change behavior in comparison to control participants. Additional gains in these two outcomes were realized after mentors completed *both* META components; this is consistent with the greater dose of training received, the more activity-focused nature of the face-to-face sessions, and the opportunity those sessions offered for learning about other participants’ mentoring experiences. Notably, the one knowledge item that did not significantly increase until posttest 2 was “Knowledge of specific strategies I can apply to maintain effective relationships and address challenges that might arise with my mentee.” This item involves learning how to skillfully apply strategies in potentially difficult mentoring situations, which we expect would be best enhanced by active engagement with others in a workshop setting.

At 3-month follow-up, META participants reported significant improvements in several skill areas in comparison to controls. Because we used different skills measures than those used to evaluate the 8-hour *EM* curriculum (26-item MCA), we cannot directly compare our data to that earlier work. However, since completion of the pilot, we have acquired data from two new META cohorts (from 2016 and from 2018), whose evaluation did include the MCA. For these mentors (total *n* = 31), the mean change in retrospective pretest to posttest MCA composite score (potential score range of 1–7) was +0.91 (*P* < 0.001, two-tailed *t*-test for paired samples). This improvement compares favorably to the mean MCA composite score gain of +0.70 reported previously [25], indicating that we can achieve comparable impact with our slightly shorter hybrid variation. Faculty in these more recent META cohorts also affirmed the synergy between the online and in-person components that we observed in the pilot, with 85% agreeing or strongly agreeing that the online module “helped prepare them to engage in the facilitated group sessions” and 82% agreeing or strongly agreeing that the facilitated group sessions “successfully built upon – and allowed me to apply – the fundamental knowledge I gained from the online module.”

We were encouraged by the magnitude of group differences in mentors’ self-appraised knowledge and score gains, which for statistically significant items ranged from 0.78 to 1.35 for knowledge and from 0.59 to 1.00 for skills (on our 1–5 scale). That said, measures of self-reported learning can be difficult to interpret in terms of practical relevance and are inherently limited by the reliability of respondents to accurately assess their own knowledge and skills. Coupled with these gains was the mentors’ ability to articulate specific behavior change intentions or actions that resulted from their engagement in the training (*OPM* alone and the full META). This is perhaps the most compelling indictor of the training’s impact. Last, it is worth noting that in the multisite RCT of the *EM* curriculum on which the in-person component of the META is based, the greater skill gains and behavior changes of intervention mentors compared to controls were confirmed by quantitative and qualitative assessments provided by their mentees [25].

Although the workshop component of the META curriculum has been well studied, this is the first publication to report outcomes data for *OPM*. Mentors in our pilot who engaged with the online, interactive material in *OPM* reported greater knowledge gains than mentors who received only a written summary of its core content. For over 40% of mentors, the online training was sufficient to prompt an intention to make changes to their current mentoring practices. These promising results support the applicability of *OPM* as a viable standalone curriculum, such as within a specific group or program when application of the synchronous group model is not feasible or as an individual professional development activity. Asynchronous, self-paced, online training has the potential to reach wide audiences and offer a higher degree of quality control than instructor-led trainings. At our institution, we require *OPM* completion by faculty who mentor trainees in our CTSI education programs. Additionally, T32 predoctoral and postdoctoral training program directors are leveraging the module to help support the NIH requirement for mentor training. At the University of Wisconsin-Madison’s Institute for Clinical and Translational Research, *OPM* is offered as a complement to face-to-face mentor training or as a substitute for those who cannot attend in-person sessions.

Since its launch, *OPM* has included a voluntary feedback survey, but response rates were low. In late 2016, we replaced the survey with a more refined evaluation that includes common metrics for “low dose” (<4 hours) mentor trainings offered by the National Research Mentoring Network (NRMN) [39]. We have been encouraging users to complete the survey by offering a downloadable certificate of course completion upon submission. Consequently, we are slowly generating a larger national dataset that will allow us to learn more about the module’s impact as a solo training element. This work is in progress, but preliminary data show that 105 of the first 120 survey respondents (87.5%) reported making or planning to make changes in their mentoring practices as a result of the online training. For this same sample, we found statistically significant increases from before to after *OPM* completion in respondents’ self-ratings for two variables: overall mentoring quality and confidence to mentor effectively. A complete manuscript reporting on the national reach of *OPM* and evaluation data for a larger sample of its users is in preparation.

### Limitations

Limitations of our pilot must be considered. Our sample was relatively small, self-selected, and drawn from one institution – all features that could affect the generalizability of our findings. However, the sample was reasonably diverse in age, sex, school/college, research focus, training stage of mentees, and extent of previous mentor training. Future work with a larger sample across multiple sites should be conducted. The majority of our participants were assistant professors and/or faculty with 5 or less years of research mentoring experience – a group that might be more likely than other faculty participants to benefit from the META. We did not conduct subgroup analyses, given our small sample size. However, previous research on the full *EM* curriculum with a larger sample – one that was composed of predominantly associate or full professors (88%) – found that even mid- to late-career faculty derived value from the training, as exhibited by significant improvements in participants’ pre- to posttest MCA composite score regardless of rank, and by qualitative reports of behavior change [25,29].

We did not include mentees in our study, so our results are limited to mentors’ self-appraisals. Mentors were not blinded to treatment assignment, so a Hawthorne effect cannot be ruled out. Despite the minimal content offered to control mentors, several indicated an intention to change their mentoring practices at the posttest (18%) or implementation of a change at 3-month follow-up (29%). This could be due to response bias, which is a limitation of our overall study, given that participants self-selected into the trial.

Last, although the META is shorter than the original *EM* curriculum – with the enhanced flexibility of having ∼25% of its content online – the program does require a three-fourth day time commitment that some faculty may still find burdensome. In our view, this is a justifiable effort to help prepare faculty for a role as essential as mentoring the next generation of clinical and translational science researchers. Program length has not been a deterrent in our ability to recruit 15–20 faculty participants per year. The workshops can function well with as few as six to eight active participants, which is a reasonable target for smaller institutions or programs.

### Conclusions, Implications, and Future Work

Overall, our findings illustrate the value of applying asynchronous e-learning approaches in hybrid models and as solo interventions for research mentor development. Our results have relevance for institutions and programs exploring options for how to optimally implement mentor training in their settings. Interest in training customization is high, exemplified by documented variation in how *EM*-based trainings are being implemented nationally [21,22]. At www.cimerproject.org, users can now select specific *EM* modules and activities to match their topics of interest and the career stage and disciplinary background of their local audiences. Specific to hybrid offerings, we are aware of two training programs using *OPM* in unique hybrid configurations. One scenario was described by a 2-year, NIH-funded, postdoctoral program at Washington University in Saint Louis [40]. Mentors in this program are required to complete a three-stage training sequence over 4 months consisting of *OPM* (asynchronous, self-paced) and two 1-hour group sessions facilitated by a mentoring consultant (one conducted virtually and focused on aligning expectations, a second conducted in person and focused on effective communication). At the Howard Hughes Medical Institute, *OPM* is embedded into an extensive mentor training program (30 hours total) required for advisors of doctoral students participating in the prestigious Gilliam Fellowship program [42,44].

Interest in different approaches to research mentor training (e.g., different delivery modalities, dosages, and content) will likely increase as more institutions invest in mentor training as a core professional development activity and as more knowledge is acquired about the attributes that make research mentoring relationships effective [43]. With the latter in mind, NRMN investigators and their partners have been developing and testing new, highly targeted training models. These include a full day, in-person curriculum focused on culturally aware mentoring [20] and another on preparing mentors to promote their mentees’ research self-efficacy [44].

Encouraged by our early evaluation results and expanding pool of registrants for *OPM*, we have developed a companion version of the module that is targeted to research mentors of undergraduate students (beta testing planned for Spring 2019). We are also finalizing an updated version of the original *OPM*. We streamlined content in *OPM 2.0* to reduce the amount of required reading and offer a more selective set of supplemental resources. We also introduced new reflection questions to encourage more real-time application of learning to practice. These questions could serve as potential at-home assignments, with answers brought to a face-to-face setting for discussion with other mentors, as was done for a different hybrid training approach implemented at the University of Rochester Clinical and Translational Science Center [16]. Additionally, we included new images and content to reinforce the importance of cultural awareness in mentoring, and introduced material on mentors’ roles in attending to psychosocial factors (such as research self-efficacy) that can influence mentees’ interest and persistence in STEM disciplines. *OPM 2.0* will be publicly available in mid-2019.

## Supplementary material

For supplementary material accompanying this paper visit https://doi.org/10.1017/cts.2019.368. click here to view supplementary material
