# Supplementary material for: A randomized controlled pilot study of the University of Minnesota mentoring excellence training academy: A hybrid learning approach to research mentor training
Source: J Clin Transl Sci. 2019 Jul 18;3(4):152–64. doi: 10.1017/cts.2019.368 (PMC6799418; doi:10.1017/cts.2019.368)
Supplement: Supplementary file 1 [file S2059866119003686sup001.pdf]

Mentoring Tips Sheet
AHC Mentorship Excellence Training Academy

UNIVERSITY OF MINNESOTA
Driven to Discover℠

1. **Communicate to mentees the importance of mentoring to their development as researchers and professionals in their field.**

   <u>Definition of *Mentoring*</u>

   Mentoring is a collaborative learning relationship that proceeds through purposeful stages over time and has the primary goal of helping a mentee to acquire the essential competencies needed for success in a research (or other) career.

   <u>Potential Benefits For Mentees Include Increases In:</u>

   - Job satisfaction
   - Career self-efficacy
   - Research productivity
   - Career satisfaction and commitment
   - Socialization to the profession and local environment

2. **Mentoring can take many forms, each of which has its own benefits and challenges.**

   <u>Examples of Mentoring Models</u>

   - *Traditional mentoring*: An aspiring junior researcher is paired with a senior investigator (or team of senior investigators).
   - *Group mentoring:* A small number of experienced senior investigators collectively serve as mentors for a larger cohort of junior researchers.
   - *Peer mentoring:* A pair or small group of researchers, all at similar career stages, gather for the purpose of mutual career development and support.

3. **The roles and responsibilities of a mentor fall into two general domains: career-enhancing and psychosocial. Mentoring in both domains is critical to a mentee's development.**

   <u>Career-enhancing Roles</u>: Mentors are focused on the external performance of mentees, specifically their professional and intellectual development. Examples of mentor responsibilities in this domain:

   - Offering guidance in research (planning, execution, and dissemination stages of projects)
   - Enhancing teaching practices
   - Developing career management skills (applying for positions, advancing in a career)
   - Identifying opportunities for professional development
   - Fostering independence
   - Being an advocate
   - Brokering opportunities

   <u>Psychosocial Roles</u>: Mentors are focused on shaping mentees' internal values and attitudes that will contribute to their success in the profession. Examples of mentor responsibilities in this domain:

   - Clarify professional identity
   - Model the behaviors, attitudes, values of the profession
   - Provide encouragement
   - Enhance confidence

4. Because mentoring is a "two-way street," it is important to discuss with mentees *their* roles and responsibilities for creating an effective mentoring relationship. Some examples follow.

Preparing for Mentoring

- Clarify your, and your mentor's, values and preferred work style
- Identify your short- and long-term career goals
- Clarify your professional development needs
- Identify specific objectives for the mentoring relationship

Choosing a Mentor

- Recognize that one mentor may not fit all of your needs
- Assess the fit between you and the potential mentor

Cultivating the Mentoring Relationship

- Discuss and align your and your mentor's expectations for the relationship
- Take initiative and follow through on commitments
- Share and be receptive to constructive feedback
- Communicate well
- Respect your mentor's time

5. A formal mentoring relationship generally consists of four phases:

1. Preparing: Self-reflect and determine the fit between mentor and mentee
2. Negotiating: Set expectations for the relationship; outline a vision, goals, strategies, and a timeline for the mentee's career advancement and mentor's roles in supporting these; use this information to create an individual development plan (IDP)
3. Enabling: Implement the mentoring plan, meet regularly, evaluate progress towards goals, address emergent challenges and opportunities
4. Closing: Conclude or prepare for a next iteration of the mentoring relationship

6. The ability to build healthy mentoring relationships requires a mentor to use a number of personal attributes and proactive strategies, such as the following:

- Appreciate individual differences in mentees, with an openness to adapting one's mentoring approaches
- Increase awareness of cultural diversity and its impact on mentoring
- Communicate openly and often
- Use appropriate self-disclosure to share one's experiences, including failures
- Establish trust
- Create and respect boundaries with mentees in areas such as time, confidentiality, and respect.
- Align expectations
- Provide a mixture of support, challenge, and vision
- Employ self-awareness and engage in self-reflection about one's mentoring practices
